# Differentiation of Normal and Radioresistant Prostate Cancer Xenografts Using Magnetization Transfer-Prepared MRI

**DOI:** 10.1038/s41598-018-28731-0

**Published:** 2018-07-11

**Authors:** Wilfred W. Lam, Wendy Oakden, Leedan Murray, Jonathan Klein, Caterina Iorio, Robert A. Screaton, Margaret M. Koletar, William Chu, Stanley K. Liu, Greg J. Stanisz

**Affiliations:** 1 Physical Sciences, Sunnybrook Research Institute, Toronto, Ontario Canada; 2 Medical Biophysics, University of Toronto, Toronto, Ontario Canada; 3 Radiation Oncology, University of Toronto, Toronto, Ontario Canada; 4 Biological Sciences, Sunnybrook Research Institute, Toronto, Ontario Canada; 5 Biochemistry, University of Toronto, Toronto, Ontario Canada; 6 Radiation Oncology, Sunnybrook Health Sciences Centre, Toronto, Ontario Canada; 7 Neurosurgery and Paediatric Neurosurgery, Medical University of Lublin, Lublin, Poland

## Abstract

The ability of MRI to differentiate between normal and radioresistant cancer was investigated in prostate tumour xenografts in mice. Specifically, the process of magnetization exchange between water and other molecules was studied. It was found that magnetization transfer from semisolid macromolecules (MT) and chemical exchange saturation transfer (CEST) combined were significantly different between groups (*p* < 0.01). Further, the *T*_2_ relaxation of the semisolid macromolecular pool (*T*_2,B_), a parameter specific to MT, was found to be significantly different (*p* < 0.01). Also significantly different were the rNOE contributions associated with methine groups at −0.9 ppm with a saturation *B*_1_ of 0.5 µT (*p* < 0.01) and with other aliphatic groups at −3.3 ppm with 0.5 and 2 µT (both *p* < 0.05). Independently, using a live-cell metabolic assay, normal cells were found to have a greater metabolic rate than radioresistant ones. Thus, MRI provides a novel, *in vivo* method to quantify the metabolic rate of tumours and predict their radiosensitivity.

## Introduction

Prostate cancer is the most prevalent non-skin cancer in men and one of the leading causes of cancer death. Both surgery and radiotherapy are well established modalities used in its treatment. Unfortunately, almost one-third of high-risk prostate cancer patients develop recurrence following external beam radiation treatment as assessed by rising prostate-specific antigen levels^1^. Recurrent tumours, which have survived radiation (i.e., radioresistant tumours), tend to display an aggressive phenotype including increased proliferation, clinically manifest as larger tumours that are typically associated with lymph node metastases, and generally have a worse prognosis^2–7^. Indeed, up to a third of patients with recurrent prostate cancer will die from their cancer^8,9^.

We believe that development of improved detection of prostate cancer radioresistance is essential for further improving patient outcomes. The non-invasive detection of radioresistant prostate cancer through quantitative MRI will allow rapid and tailored treatment decisions to be made, such as the addition of radiosensitizers or, alternatively, the use of surgical resection in place of radiation treatment.

Previous studies have assessed tumour response to therapy using diffusion-weighted MRI^10,11^ (DW-MRI), dynamic contrast-enhanced MRI^12^ (DCE-MRI), and positron emission tomography^13,14^ (PET). Some of these techniques were able to detect radioresistance as early as a few weeks or a month. Confounding factors include sensitivity to multiple tissue features such as microstructural geometry and permeability which may not be specific to radiation resistance^11,15^. Furthermore, DCE-MRI requires the injection of contrast agent^16^, and PET exposes the patient to ionizing radiation and has a limited resolution^17^.

Chemical exchange saturation transfer^18^ (CEST) and the relayed nuclear Overhauser effect^19^ (rNOE) are promising magnetic resonance contrast mechanisms that are sensitive to metabolism^20^, can provide contrast without an exogenous contrast agent, and can potentially predict tumour response before treatment^21^. They are measured using magnetization transfer-prepared pulse sequences, which are sensitive to the exchange of magnetization between the hydrogen nuclei in water and other molecules. CEST is the physical exchange of hydrogen atoms in chemical groups in dissolved proteins (e.g., amide^22^, amine^18^, guanidinium^23,24^, and hydroxyl^18^) with water. rNOE is the exchange of magnetization intramolecularly through space between chemical groups (e.g., methine^25,26^ and other aliphatic^19^) with other hydrogen nuclei, which then undergo CEST^27^. In addition to CEST and rNOE, magnetization transfer-prepared pulse sequences can measure the exchange of magnetization between semisolid macromolecules (mostly lipid bilayers) and water, which is termed magnetization transfer^28,29^ (MT).

In magnetization transfer-prepared pulse sequences, magnetization is reduced by a radiofrequency saturation pulse (of amplitude *B*_1_) across the various frequencies of the exchanging molecules. The ratio of this reduced water signal (*S*) to the signal without saturation (*S*_0_) is calculated for each frequency offset, and from this the magnetization transfer ratio (MTR) can be calculated as: 1 – *S*/*S*_0_. In addition to the exchange rates of magnetization between hydrogen nuclei in the semisolid macromolecular, CEST, and rNOE pools with those in water, the MTR is also sensitive to the sizes and longitudinal and transverse relaxation times (*T*_1_ and *T*_2_, respectively) of each pool. It is also common to present the CEST data in a form of a “Z-spectrum”, which is a plot of measured water signal as a function of saturation pulse frequency offset (Δ*ω*) acquired over multiple excitations.

A radioresistant prostate cancer cell line has been developed to further investigate radioresistance^30^. Tumours arising from radioresistant cells are structurally similar to the parental ones and have similar standard MR properties such as *T*_1_ and *T*_2_ relaxation times and diffusion properties. We have demonstrated *in vitro* that radioresistant cells possess altered metabolism compared to normal (parental) ones and, consistent with this, discovered that they exhibit different MT, CEST, and rNOE effects *in vivo* – MRI contrasts that are sensitive to the chemical environment. In this work, we show that normal and radioresistant tumours in an animal model can be differentiated by MTR and isolate the underlying MT and rNOE contributions.

## Results

In this work, the CEST effect was measured for 7 parental and 6 radioresistant DU145 prostate tumours xenografts *in vivo*. We have also analyzed the contributions of several MRI effects to the CEST spectra which allowed us to determine which of the many MRI processes (relaxation, MT, CEST, or rNOE) are the major contributor for the observed changes between parental and radioresistant tumour xenografts.

**Figure 1:**
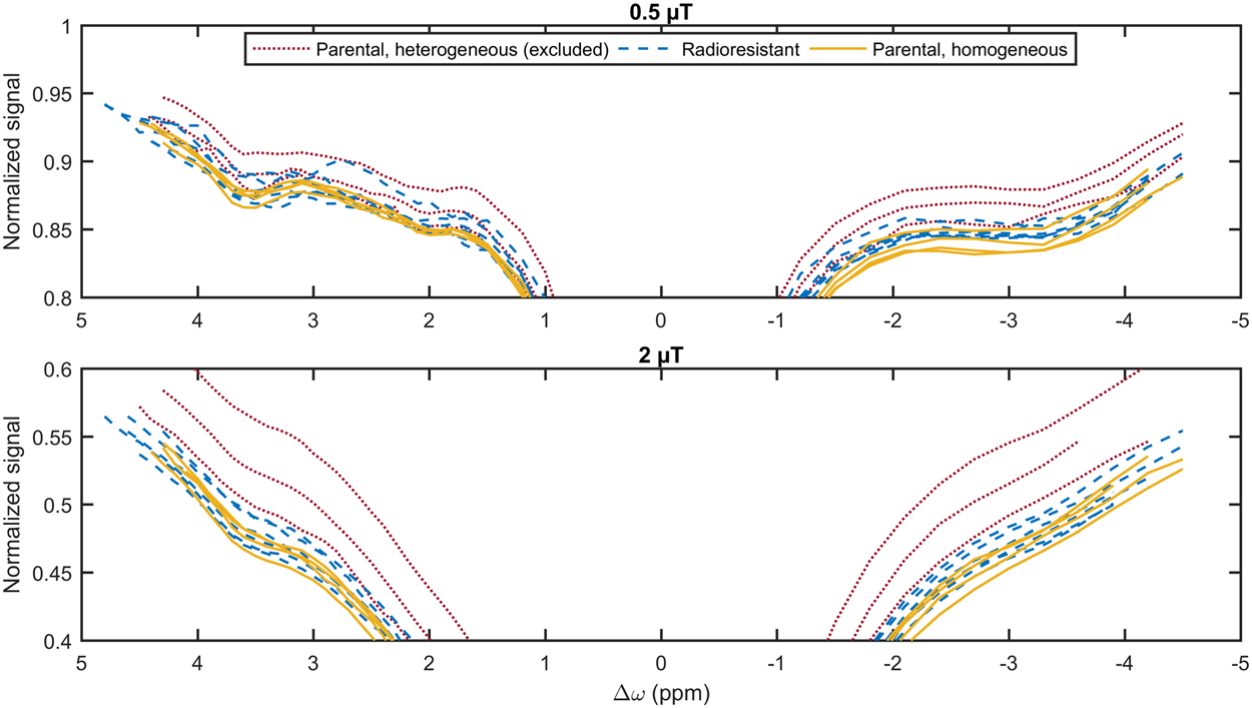
Measured Z-spectra with saturation *B*_1_s of 0.5 and 2 µT of all homogeneous and heterogeneous tumours, both derived from the parental cell line, and tumours derived from the radioresistant cell line.

In the parental group, three tumours were substantially heterogeneous on the *T*_2_-weighted image (Supplementary Fig. S1) and large regions of elevated *T*_1_ and *T*_2_ indicating significant necrosis (not shown) were seen on *T*_1_ and *T*_2_ maps. Their Z-spectra were also substantially different than those of the other parental and all the radioresistant tumours (Fig. 1). On these bases, they were excluded from further analysis.

### Z-spectrum analysis

**Figure 2:**
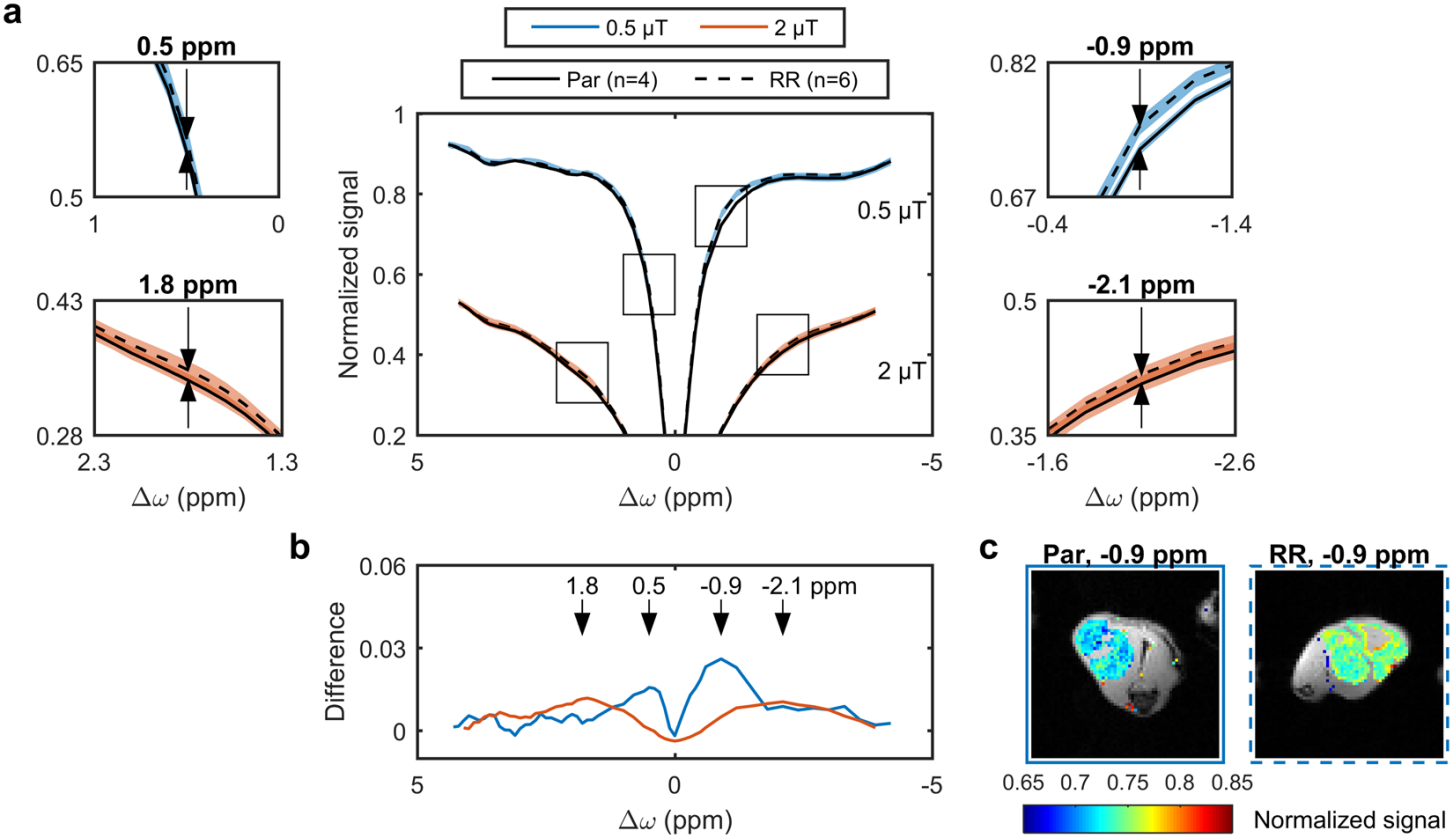
Z-spectra of parental (solid lines) and radioresistant (dashed lines) tumours. (**a**) The mean for parental (Par, *n* = 4), and radioresistant tumour (RR, *n* = 6) Z-spectra (shaded areas represent the standard deviations) with saturation *B*_1_s of 0.5 (blue) and 2 µT (orange). (**b**) Differences between Par and RR showing several maxima (arrows) per saturation *B*_1_. (**c**) Magnetization transfer-prepared images (with Rician noise bias and *B*_0_ correction) overlaid on the CEST reference images for representative tumours with a saturation *B*_1_ of 0.5 µT at a frequency offset of −0.9 ppm are also shown.

**Figure 3:**
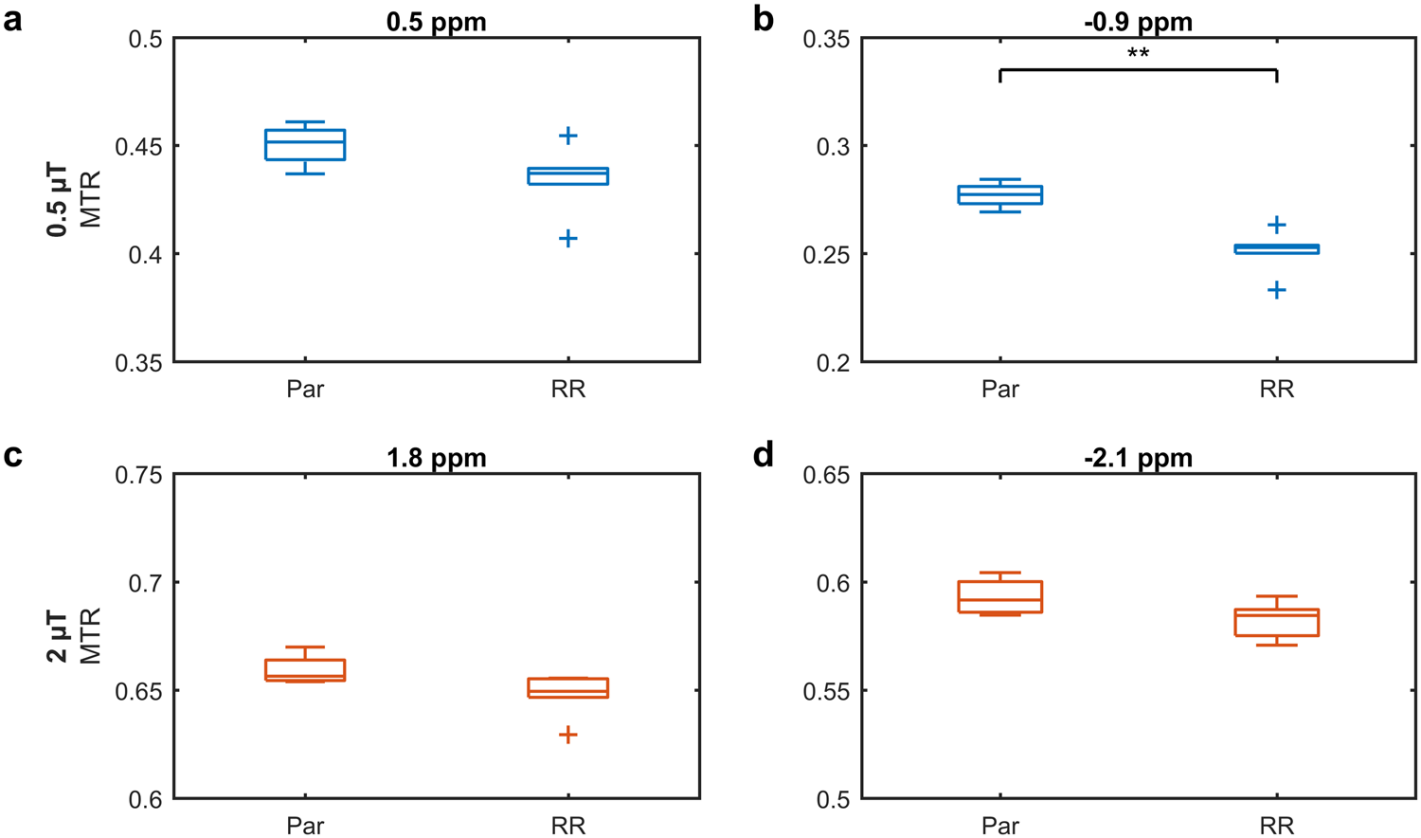
Statistical comparison of the magnetization transfer ratios (MTRs) between parental (Par) and radioresistant tumours (RR) at the offsets indicated (arrows) in Fig. 2b. **p < 0.01.

The averaged Z-spectra of the remaining homogeneous parental and radioresistant tumours are shown in Fig. 2a at saturation *B*_1_s of 0.5 (blue) and 2 µT (orange). The mean Z-spectra for parental and radioresistant tumours, although similar in shape, exhibited significant differences, which are more visible in the enlarged plots in Fig. 2a. Figure 2b shows the difference between the mean Z-spectra for two measured saturation amplitudes demonstrating the presence of several maxima per saturation *B*_1_. The MTR with a saturation *B*_1_ of 0.5 µT at Δ*ω* = −0.9 ppm (*p* = 0.002; Fig. 3b) is significantly different. CEST contrast images are in Supplementary Fig. S2. Observed *T*_1_s (from the inversion recovery images) for parental and radioresistant tumours were 2260 ± 100 and 2300 ± 70 ms, respectively, while observed *T*_2_s (from the inversion recovery images and WASSR spectrum) were 61 ± 7 and 64 ± 4 ms, respectively, neither of which was significantly different between groups. *T*_1_ and MTR histograms are in Supplementary Fig. S3. Repeatability of the MTR was found to be good (Supplementary Fig. S5a,b).

### Isolating MT, CEST, and rNOE contributions

**Figure 4:**
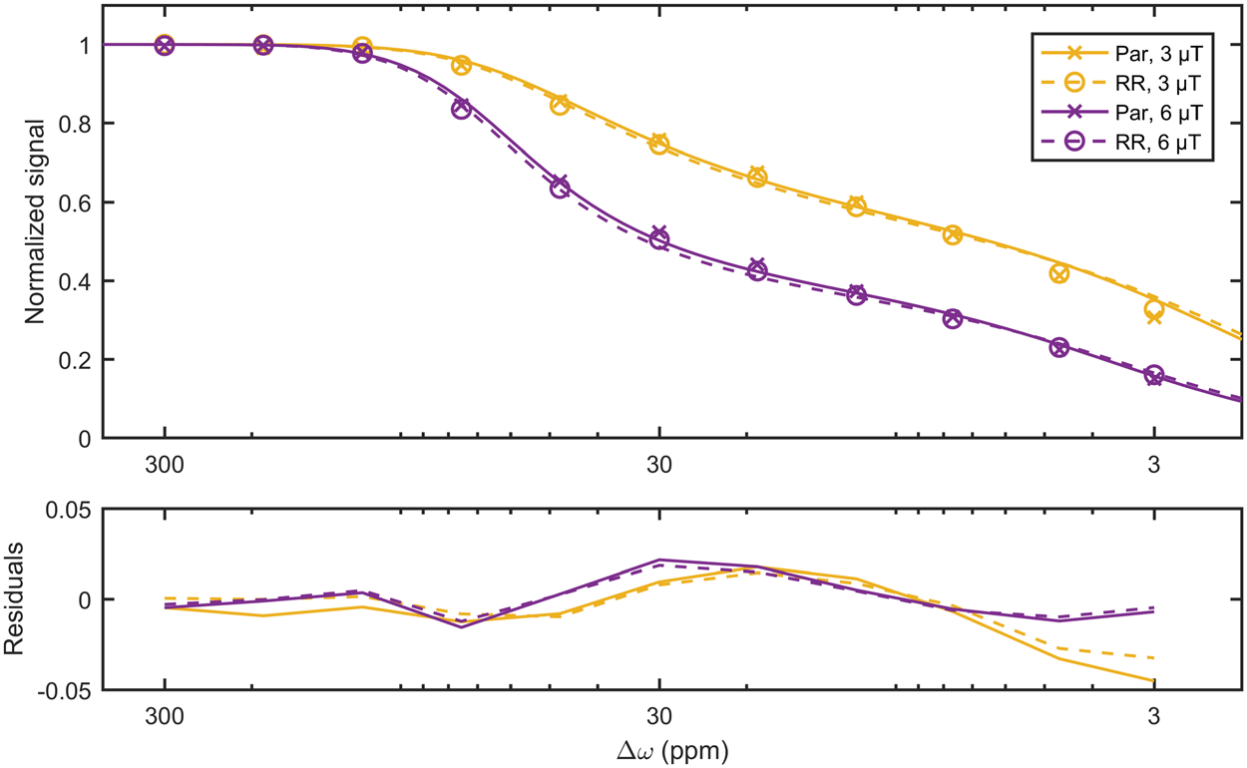
Results (lines) of simultaneously fitting the measured MT-sensitive Z-spectra (points) at saturation *B*_1_s of 3 and 6 µT to the two-pool MT model for representative parental and radioresistant tumours. Fitting residuals are also shown.

**Figure 5:**
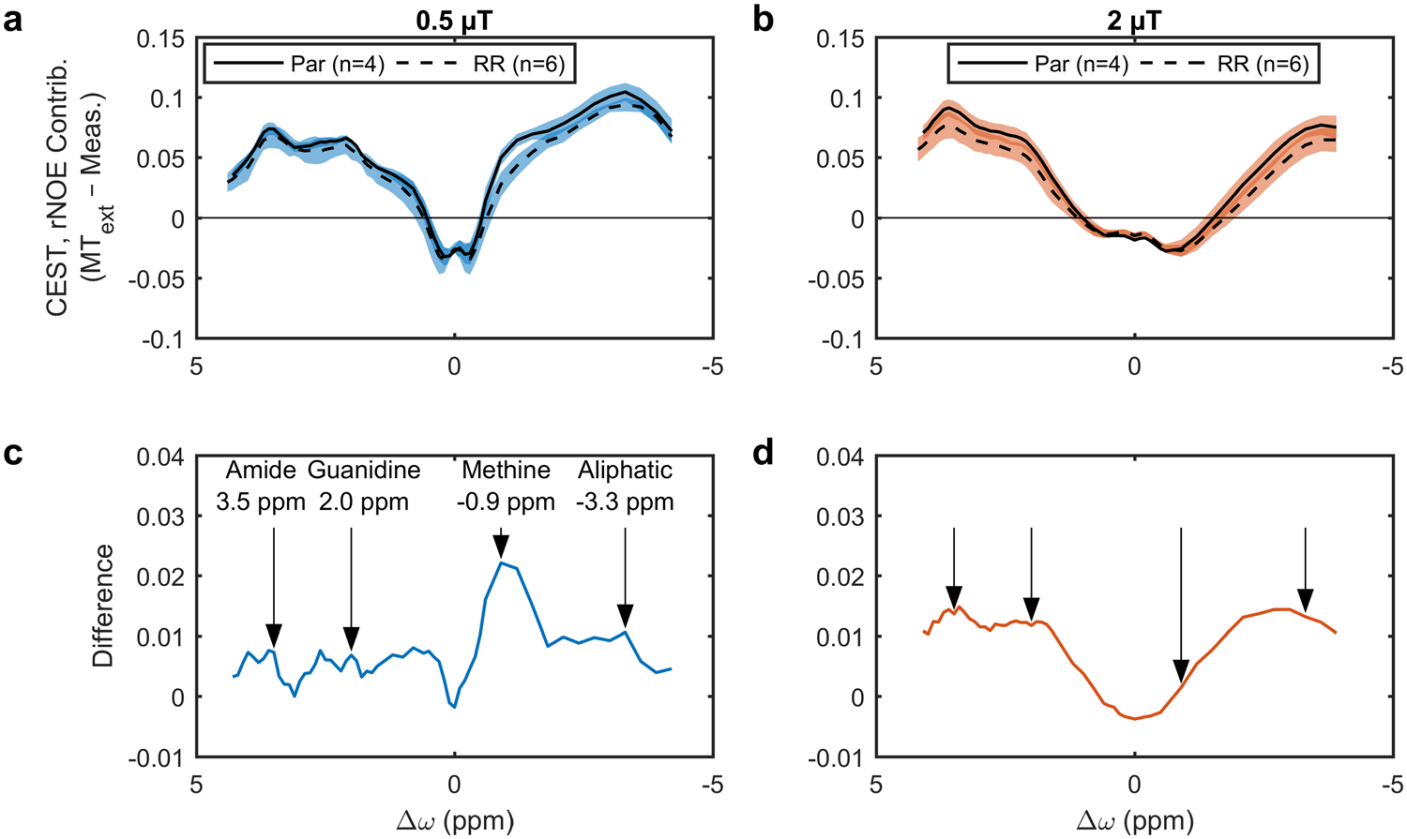
The CEST and rNOE contributions of parental (Par) and radioresistant (RR) tumours. Mean CEST and rNOE contributions to the MTR (shaded areas indicate the standard deviations), given by the difference between extrapolated semisolid molecular MT (MT_ext_) and measured Z-spectra, at saturation *B*_1_s of (**a**) 0.5 and (**b**) 2 µT and (**c**,**d**) their respective differences. Arrows indicate the commonly identified CEST and rNOE pool frequency offsets. The methine pool is not usually identified in literature because its Z-spectrum peak is close to that of water and not always distinguishable.

**Figure 6:**
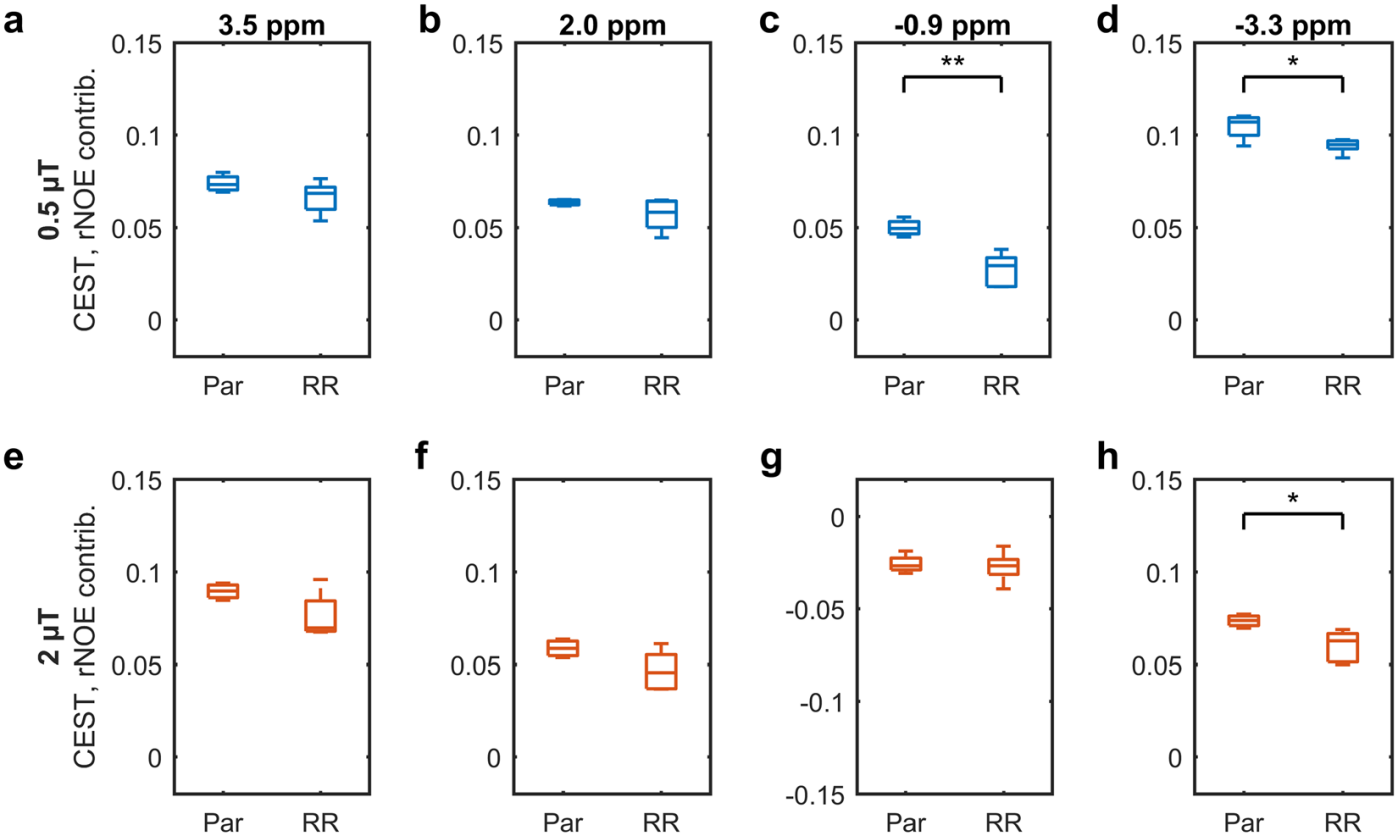
Statistical comparison of the CEST and rNOE contributions between parental (Par) and radioresistant (RR) tumours. Boxplots of the CEST and rNOE contributions to the MTR with saturation *B*_1_s of (**a–d**) 0.5 and (**e–h**) 2 µT at the frequency offsets indicated by the arrows in Fig. 5c and d. *p < 0.05. **p < 0.01.

Z-spectra for saturation *B*_1_s of 3 and 6 µT, were fitted to a two-pool MT model (Fig. 4). Table 1 shows that, of all the parameters fitted, only the *T*_2_ of the semisolid macromolecular pool *T*_2,B_ was significantly different (*p* = 0.008) between the parental and radioresistant groups. These fitted parameters were used to extrapolate the MT model Z-spectra to the CEST- and rNOE-sensitive saturation *B*_1_s of 0.5 and 2 µT. A schematic is in Supplementary Fig. S4. The difference between this modelled MT and the measured Z-spectra was calculated in order to isolate the contributions of CEST and rNOE (Fig. 5). Also significantly different are the rNOE contributions associated with methine groups at −0.9 ppm with a saturation *B*_1_ of 0.5 µT (*p* = 0.001; Fig. 6c) and with other aliphatic groups at −3.3 ppm with 0.5 µT (*p* = 0.015; Fig. 6d) and 2 µT (p = 0.018; Fig. 6h). Repeatability of the CEST and rNOE contributions was also found to be good (Supplementary Fig. S5c–f).

**Table 1:** Estimated parameters of the two-pool MT model from fitting the Z-spectra with saturation *B*_1_s of 3 and 6 µT of the parental and radioresistant tumours.

Parameter	Parental	Radioresistant	*p*-value
*R*_1,W_ (1/s)	0.43 ± 0.02	0.42 ± 0.01	0.419
*T*_2,W_ (ms)	58 ± 6	61 ± 4	0.347
*R* (Hz)	36 ± 2	37 ± 7	0.851
*M*_0,B_ (%)	3.0 ± 0.2	3.3 ± 0.2	0.102
***T***_**2**, **B**_ **(µs)**	**8**.**44** ± **0**.**29**	**8**.**02** ± **0**.**05**	**0**.008**

This model has one calculated parameter: *R*_1_ of the water pool (*R*_1,W_) and four free parameters: *T*_2_ of the water pool (*T*_2,W_), exchange rate from the semisolid macromolecular pool to the water pool (*R*), initial magnetization of semisolid macromolecular pool (*M*_0,B_) relative to that of the water pool (defined as unity) and *T*_2_ of the semisolid macromolecular pool (*T*_2,B_). **p < 0.01.

### Oxygen consumption rate measurement

**Figure 7:**
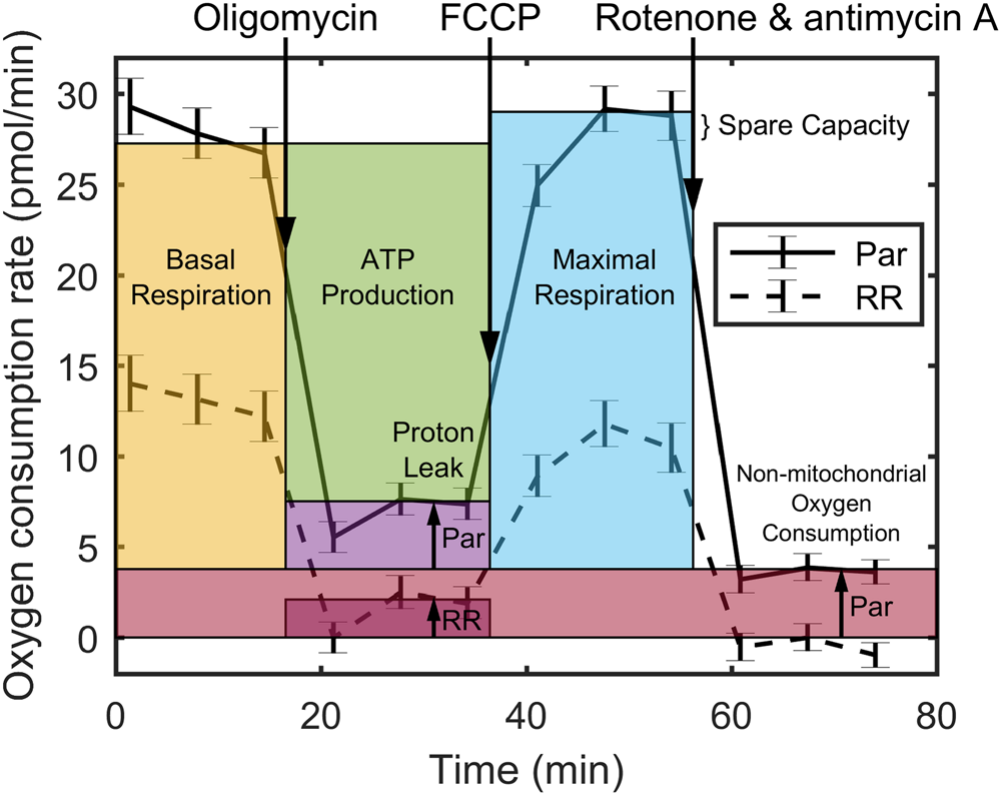
Oxygen consumption rate of parental (Par) and radioresistant (RR) cells. Mean oxygen consumption rate (error bars are standard error) modulated by serial injections (downward arrows) of oligomycin, which inhibited ATP synthase, but allowed the facilitated diffusion of protons or “proton leak” across the inner mitochondrial membrane to continue; *p*-trifluoromethoxy carbonyl cyanide phenylhydrazone (FCCP), which maximized oxygen consumption; and rotenone and antimycin A, which stopped all mitochondrial respiration.

We also measured modulation of the oxygen consumption rate of parental and radioresistant cells (Fig. 7) using an extracellular flux analyzer. The sections of the plot between injections indicate, from left to right, that parental cells have higher basal respiration, proton leak, maximal respiration, and non-mitochondrial respiration than radioresistant cells. Overall, this demonstrates that parental cells have a greater metabolic rate than radioresistant cells (i.e., they consume more oxygen in the basal state and have a larger spare respiratory capacity), which provides biological correlation with our observed CEST and rNOE findings.

## Discussion

We have demonstrated that radioresistant and parental prostate tumour xenografts can be differentiated using only the magnetization transfer ratio (MTR) at a single, low saturation *B*_1_ (Fig. 3), which is a relatively simple MRI measurement. The acquisition of two MT-sensitive Z-spectra (acquired at high saturation amplitudes) and a *T*_1_ map, permitted fitting of MT parameters which showed the significant difference in *T*_2,B_ between radioresistant and prostate tumour (Table 1). Additional acquisition of full Z-spectra at low saturation *B*_1_s (0.5 and 2.0 µT), in combination with the modelled MT contribution extrapolated to these *B*_1_s, separated out the relative contributions of CEST and rNOE in both tumour types (Fig. 5). Tumours were automatically segmented by thresholding a *T*_2_ map calculated from the *T*_1_ map and a WASSR Z-spectrum using Eq. 2.

Both the semisolid macromolecular and aliphatic rNOE pools contributed to the difference in MTR between the two tumour groups (Table 1 and Fig. 6). The ability to distinguish the contributions from the semisolid macromolecular and aliphatic rNOE pools provided additional specificity. The simple MTR measure was also affected by the direct water saturation^27,29^, which is a function of *T*_1_ and *T*_2_. However, the relaxation values of parental and radioresistant tumours were similar indicating that the differences in the MTR originated primarily from semisolid macromolecular MT and rNOE.

This dual contribution to the MTR signal was also the reason we do not use MTR asymmetry^31^ (MTR_asym_), a common metric to identify CEST contrast. It is calculated by subtracting the Z-spectrum at positive offsets from the corresponding negative offsets. MTR_asym_ combines the contrast from CEST and rNOE, which in this case actually decreased the difference between parental and radioresistant groups.

The observation that radioresistant tumours exhibited lower CEST and rNOE (Fig. 5a) was consistent with the *in vitro* observation that radioresistant tumour cells had lower metabolism than the parental ones (Fig. 6). Our previous clinical studies have also found significant pre-treatment rNOE differences between gliomas responsive and non-responsive to stereotactic radiosurgery^21^ and MT, CEST, and rNOE differences between radiation necrosis and tumour progression in brain metastases^32^.

Of the semisolid macromolecular MT parameters, the only one showing significant difference was the semisolid macromolecular relaxation time *T*_2,B_. It was slightly lower in radioresistant tumours (8.02 ± 0.05 µs) than parental (8.44 ± 0.29 µs). *T*_2,B_ has been found to vary with tissue type^33^, and changes in *T*_2,B_ have also been attributed to the degree of cross-linking of molecules (i.e., in polyacrylamide gels with *T*_2,B_ varying inversely with rigidity^34^). Decreased *T*_2,B_ has been observed in spinal cord neuropathy where it was interpreted as a change in macromolecular structure of myelin^35^. We believe that this is the first observation of this phenomenon in cancer tissue where it may indicate slight changes in the rigidity of cell membrane lipids which are believed to be responsible for MT effects in tumours.

Our isolation of the CEST and rNOE contributions differed from the extrapolated semi-solid magnetization transfer reference (EMR) method by Heo *et al*.^36^ in several respects. First, we acquired Z-spectra at logarithmically spaced frequency offsets from 300 to 3 ppm, whereas Heo *et al*. acquired Z-spectra with linearly spaced offsets from 21 to −21 ppm, excluding the range from 7 to −7 ppm to avoid CEST and rNOE effects. Note that the semisolid macromolecular MT Z-spectrum has a feature around 50 ppm^37^ that necessitates data collection beyond 21 ppm. Second, we simultaneously fitted the Z-spectra and *T*_1_ map to the two-pool MT model, whereas Heo *et al*. fitted the Z-spectra alone to yield parameters lumped together with the *T*_1_ of the water pool and then isolated the parameters using the *T*_1_ map. We feel that simultaneous analysis of all the data allowed the fitting algorithm to better constrain the parameter estimates. Third, after subtracting the semisolid macromolecular MT contribution from the low *B*_1_ Z-spectra, we compared the CEST- and rNOE-only MTRs, whereas Heo *et al*. fitted the Bloch–McConnell^38^ magnetization exchange equations to estimate the pool sizes, exchange rates, and *T*_2_s of the amide (3.5 ppm) and rNOE pools (−2.5 to −5 ppm). Each has a unique advantage. The benefit of fitting the Bloch–McConnell equations is that the parameters are *B*_1_-independent. The benefit of comparing CEST- and rNOE-only MTRs is that it does not require the collection of full Z-spectra at lower *B*_1_. We attempted fitting of the Bloch–McConnell equations with amide (3.5 ppm), guanidinium (2 ppm), and rNOE pools (−3.3 ppm) to the CEST- and rNOE-only Z-spectra as well as all the data (all Z-spectra and the *T*_1_ map), but there was structure in the residuals and this was left to future work. Some misfitting is also seen in the work by Heo *et al*. (Fig. 3, solid lines with a saturation *B*_1_ of 0.5 µT), possibly due to the lack of a pool in their model at 2 ppm.

Another challenge to the modelling and interpretation of CEST and rNOE data is that our understanding of the rNOE contributions is limited. rNOE is thought to be composed of several peaks corresponding to different aliphatic groups. However, even at the extremely high field of 21.1 T^39^ it appears as one broad peak, instead of multiple peaks. Phantom studies would help, but it is difficult to produce a simple phantom with an aliphatic rNOE pool. *Ex vivo* rNOE has been measured using protein-free brain lipids extracted from mice, but not in synthetic liposomes^40^. It has also been studied using water-exchange (WEX) filter spectroscopy experiments, which showed that the rNOE process exchanges magnetization with water over hundreds of milliseconds, much slower than the CEST exchange rate (<100 ms)^19^.

The *T*_2_-weighted structural image gave better contrast between muscle, tumour, and liquid voxels, which was needed for accurate tumour segmentation. However, a *T*_2_ map would have the desirable property over a *T*_2_-weighted image of not being TR- and TE-dependent. Unfortunately, a *T*_2_ map was not part of the original imaging protocol and was generated from the *T*_1_ and WASSR data instead, which is not conventionally done. In our opinion, a more standard CPMG-calculated^41,42^ *T*_2_ map would be free of any potential cumulative errors from the *T*_1_ and WASSR scans and does not take long to acquire for a single slice. A CPMG sequence will be included in future studies.

There is likely a difference in pH between the two tumours types to which CEST may be sensitive. Although we have not assayed pH or reactive oxygen species levels in our tumours to date, based on the reduced oxygen consumption rate of the radioresistant cells relative to parental cells, this could translate to reduced hypoxia and less acidosis in the radioresistant cells. It is also possible that, given tumour heterogeneity, the exported lactate (resulting from a reduction in oxygen consumption and conversion of pyruvate to lactate by lactate dehydrogenase) is used as fuel by neighbouring cells, so an increase in acidosis may not be seen. This is further corroborated by the following CEST-derived metric. Ward and Balaban^43^ demonstrated that pH is a function of the expression:

1 $${\rm{ratio}}=\frac{{M}_{z}^{\mathrm{Site}\,2}{({M}_{0}-{M}_{z})}^{\mathrm{Site}1}}{{M}_{z}^{\mathrm{Site}\,1}{({M}_{0}-{M}_{z})}^{\mathrm{Site}2}}$$

where *M*_z_ is the measured CEST contrast, *M*_0_ is the contrast in the absence of radiofrequency saturation or under control saturation, and the sites refer to different chemical groups that are saturated. McVicar *et al*.^44^ showed that, when Site 2 is assigned to amine (2.75 ppm) and Site 1 to amide (3.5 ppm), Eq. 1 is inversely proportional to pH. Desmond^45^ reported a similar finding with guanidinium (2 ppm) in place of amine. Eq. 1 was applied to our Z-spectrum measurements with saturation *B*_1_s of 0.5 and 2 µT with Site 2 assigned to guanidinium and Site 1 to amide and *M*_0_ defined as unity. Eq. 1 had a lower value in the radioresistant tumours compared to parental (Supplementary Fig. S6), indicating that the radioresistant tumours may have a higher pH than parental ones. However, the results are not statistically significant, nor could they be translated to pH values without a calibrated standard curve. Additionally, we acknowledge that since we did not measure tumour pH and lactate, the exact mechanism remains to be elucidated.

For applications *in vivo*, the acquisition of all the Z-spectra, as performed in this work, would be too time consuming. Fortunately, to arrive at the conclusions presented requires much less data collection: two Z-spectra with high saturation *B*_1_ (3 and 6 µT) in order to extrapolate the semisolid macromolecular contribution to low saturation *B*_1_ (≈ 0.5 µT), a partial Z-spectrum with the lower saturation *B*_1_ around −0.9 ppm frequency offset to isolate the methine rNOE contribution and/or −3.3 ppm to isolate other aliphatic rNOE contributions or both (as single measurements at each of these offsets would be insufficient in the presence of *B*_0_ inhomogeneity), a WASSR scan for *B*_0_ correction, and a *T*_1_ map for two-pool MT model fitting.

We chose to use magnetization transfer-prepared FLASH to ensure that the net magnetization is in steady state with respect to saturation (i.e., saturation duration > 5 × *T*_1_). This simplifies quantitative MT modelling and future work in quantitative CEST because the effective saturation duration is 16 s (32 phase-encoding lines to reach the centre of k-space × 500 ms TR). Note that this should not to be confused with equilibrium magnetization reached after repeated TRs. In clinical imaging, faster readouts are used, where magnetization is not in steady state with respect to saturation^46^ and measurements at fewer frequency offsets are made, but these may be necessary trade-offs. Hardware limitations include lower RF amplitude and RF duty cycle, which may limit the saturation duration, require pulsed saturation (complicating modelling), and add dead time in each TR. All of this reduces image contrast relative to that from experiments on animal scanners. The clinical research magnetization transfer-prepared protocol for head imaging in our lab consists of single slice MT-prepared turbo field echo sequence with a saturation *B*_1_ of 0.5 µT made of four 242 ms block pulses, at 64 frequency offsets and 5 reference scans (1 mm × 1 mm in-plane resolution, 1.5 mm through-plane; 10 min in total) and one average each with *B*_1_s of 3 and 5 µT at 11 frequency offsets (3.5 min in total); WASABI^47^ for *B*_0_ and *B*_1_ mapping (1 min); a series of low flip angle fast field echo scans for *T*_1_ mapping (2 min); and a CPMG sequence for *T*_2_ mapping (1.5 min) for a total of 18 min. The trade-off between frequency offsets and number of slices for increased coverage should also be considered.

## Methods

### Animal model

Two cell lines were used in this study: a parental line, DU145 human prostate adenocarcinoma (ATCC, Manassas, VA; denoted “Par”) and a radiation-resistant line^30^ (denoted “RR”) generated by treatment of parental cells with radiation mimicking a clinical treatment schedule. Approximately 3 × 10^6^ cells mixed in a 1:1 ratio with growth factor reduced Matrigel matrix (BD Canada, Mississauga, ON) were injected in the right hind limbs of female athymic nude mice (Charles River Canada, Saint-Constant, QC) and allowed to grow into tumours (*n*_Par_ = 7, and *n*_RR_ = 6). Tumours were allowed to grow until they reached a volume of at least 100 mm^3^ measured using calipers every 1–4 days and calculated using the formula volume = length × width^2^/2 and until they were at least 34 days post-injection to allow time for cell differentiation. All experimental procedures in this study were approved by the Animal Care Committee of the Sunnybrook Research Institute, which adheres to the Policies and Guidelines of the Canadian Council on Animal Care and meets all the requirements of the Animals for Research Act of Ontario and the Health of Animals Act of Canada.

### Magnetic resonance imaging

All tumours were scanned at 7 T (BioSpec 70/30 USR with BGA-12SHP gradients running ParaVision 6.0.1, Bruker BioSpin, Billerica, MA) using a 86 mm inner diameter volume coil (T12053V3) for transmit and a 20 mm diameter loop surface coil (T115534) for receive. A 2D axial *T*_2_-weighted rapid acquisition with refocused echoes^48^ (RARE; TR = 2500 ms; TE = 9.2 ms; FOV = 20 mm × 20 mm × 7.5 mm; slice thickness = 0.5 mm; matrix = 128 × 128; RARE factor = 12; bandwidth = 33 kHz; averages = 4; 6 min, 40 s) was used for prescribing the slice of interest, chosen to be at the thickest point of the tumour. *B*_0_-map-based shimming (map shim) was performed in an ellipsoidal volume enclosing the tumour in the slice of interest. Flip angle scale factor maps (Supplementary Fig. S7) were calculated^49^ for four mice using a series of 3D high flip angle FLASH scans and the *T*_1_ map for the slice of interest and the flip angle in the tumour region of interest (ROI) was found to be within 6% of nominal. Thus, *B*_1_ correction was deemed unnecessary, given the time constraints. Z-spectra (plots of water signal normalized by a reference signal vs saturation frequency offset, where water = 0 ppm) composed of single slice images were calculated from magnetization transfer-prepared (block saturation pulse; duration per k-space line = 490 ms) fast low angle shot^50^ (FLASH; TR = 500 ms; TE = 3 ms; flip angle = 30°; FOV = 20 mm × 20 mm × 1 mm; matrix = 64 × 64; bandwidth = 50 kHz) as in our previous work^51^. The cumulative saturation time when acquiring the centre of k-space is ≈ 16 s. Five Z-spectra were acquired: two spectra sensitive to the direct water saturation effect (DE), CEST, and MT contributions with radiofrequency saturation amplitudes, *B*_1_s, of 0.5 and 2 µT at 66 frequency offsets Δ*ω* (= *ω* − *ω*_0_, where *ω* is the saturation frequency and *ω*_0_, the water resonance frequency) between ±5 ppm; two spectra mainly sensitive to the DE and magnetization transfer from semisolid macromolecules (MT) with saturation *B*_1_s of 3 and 6 µT at 11 logarithmically spaced offsets between 300 and 3 ppm; and one DE-sensitive water saturation shift referencing^52^ (WASSR) spectrum with a saturation *B*_1_ of 0.1 μT at 21 linearly spaced offsets between ±0.5 ppm. After every five Z-spectrum measurements, a reference scan at an offset of 667 ppm was interleaved for baseline correction. In addition, two initial and one final reference scans were acquired for each Z-spectrum. The scan time for the Z-spectra including reference scans with saturation *B*_1_s of 0.5 and 2 µT was 44 min/spectrum; 3 and 6 µT, 8.5 min/spectrum; and 0.1 µT, 15 min. Five inversion recovery RARE^53^ scans (TR = 10,000 ms; TE = 6 ms; TI = 30, 110, 390, 1400, 5000 ms; same FOV and matrix as FLASH; RARE factor = 4; bandwidth = 77 kHz; 2 min each) were also acquired for a *T*_1_ map. The total acquisition time including scout and shimming per animal was 2.5 h.

### Animal monitoring

Anaesthesia in the animals was induced with 5% isoflurane in oxygen flowing at 1.5 L/min. 200 µL of saline solution was injected subcutaneously at the start of the scan to maintain hydration. Monitoring was performed with a temperature probe and respiratory pillow (Small Animal Instruments, Inc., Stony Brook, NY), both placed under the belly against the skin. Heating was supplied by a water bed system (same manufacturer). Skin temperature was kept at 36 °C and the isoflurane concentration adjusted around 1.75% such that the respiratory rate was maintained around 90 breaths/min.

### Image analysis

The first reference scan of each Z-spectrum was discarded in case it was not in a steady state. The distribution of signal in a manually drawn background ROI was confirmed to be Rician and Rician noise bias correction^54^ was applied to all Z-spectra. For each animal, images were registered using a rigid body transform to the first reference image with a saturation *B*_1_ of 0.5 µT. Z-spectrum images with less than 75% of the mean signal of the reference scan were considered to have insufficient signal-to-noise ratio (SNR) for the calculation of a transform matrix and were registered using the last matrix with sufficient SNR (typically an interleaved reference scan, which were acquired frequently and had high SNR). To correct for baseline drift, the measurements of each Z-spectrum were normalized to a line fitted to the reference measurements interleaved with the Z-spectrum measurements (reference measurements are not shown). To correct for *B*_0_ inhomogeneity, which introduces a shift in the Z-spectrum along the frequency offset axis, the sum of two Lorentzians (corresponding to DE and semisolid macromolecular pools) was fitted to the Z-spectra with saturation *B*_1_s of 0.5 and 2 µT at offsets between ±0.5 ppm. The Z-spectra were re-centred to the peak position of the water-pool Lorentzian and linearly interpolated to the frequency offsets measured originally. Similarly, for the WASSR spectrum, a single Lorentzian was fitted for *B*_0_ correction (since there is negligible semisolid macromolecular MT for low *B*_1_ saturation amplitudes). This spectrum-wise *B*_0_ correction was chosen, instead of using WASSR to correct all spectra, in case *B*_0_ drifted during acquisition.

A *T*_1_ map was calculated from the inversion recovery scans by fitting to the inversion recovery RARE signal equation^53^. Then, a *T*_2_ map was evaluated from the *T*_1_ map and WASSR spectrum (Supplementary Fig. S4, upper left) using the steady-state direct water saturation signal intensity

2 $$S({\rm{\Delta }}\omega )={S}_{0}\frac{{R}_{1}[{R}_{2}^{2}+{\{{\rm{\Delta }}\omega \}}^{2}]}{{R}_{1}[{R}_{2}^{2}+{\{{\rm{\Delta }}\omega \}}^{2}]+{\omega }_{1}^{2}{R}_{2}},$$

where *R*_1/2_ = 1/*T*_1/2_ and *ω*_1_ = *γB*_1_; *γ* is the gyromagnetic ratio of the hydrogen nucleus. The tumour ROI was defined in each animal as voxels with *T*_1_ < 2600 ms to exclude liquid and *T*_2_ ≥ 45 ms to exclude muscle and thrombus (Supplementary Fig. S4, upper right). The mean signal of each tumour ROI was calculated and Z-spectra at each saturation *B*_1_ were generated. The MTR between parental and radioresistant tumour groups were compared at the offsets with the largest signal differences.

The MTR contains contributions from the water, semisolid macromolecular (MT pool), CEST, and rNOE pools and are dependent on saturation *B*_1_, so further processing was used to disentangle the source of any differences between groups. The Z-spectra with saturation *B*_1_s of 3 and 6 µT and *T*_1_ map (Supplementary Fig. S4, centre left) were fitted to a two-pool MT model^55^ with a super-Lorentzian lineshape for the semisolid pool to quantify MR parameters of the tumours that are independent of saturation *B*_1_. This model has four fitted parameters: *T*_2_ of the water pool (*T*_2,W_), exchange rate from the semisolid macromolecular pool to the water pool (*R*), initial magnetization of semisolid macromolecular pool (*M*_0,B_) relative to that of the water pool (defined as unity) and *T*_2_ of the semisolid macromolecular pool (*T*_2,B_) and one calculated parameter: *R*_1_ of the water pool (*R*_1,W_), evaluated from the *T*_1_ map and fitted parameters. These parameters, describing only the direct effect and magnetization transfer of semisolid macromolecules, were also compared between groups.

Finally, a method to isolate CEST and rNOE contributions, similar to the extrapolated semisolid magnetization transfer reference (EMR) technique^36^, was employed. Two Z-spectra containing only semisolid macromolecular MT and water contributions were forward modelled using the estimated two-pool MT model parameters for saturation *B*_1_s of 0.5 and 2 µT (Supplementary Fig. S4, centre) and experimental Z-spectra with the same saturation *B*_1_ were subtracted to isolate the CEST and rNOE contributions (Supplementary Fig. S4, bottom). The contributions are artificially negative around 0 ppm likely because the exchange of water magnetization with the semisolid macromolecular pool is overly weighted, even though it also exchanges with the CEST and rNOE pools, because the semisolid macromolecular model is fitted first. However, this does not affect the estimated contributions at the CEST and rNOE peak locations. The contributions at peak offsets of 3.5 ppm (amide; CEST), 2.0 ppm (guanidinium; CEST), −0.9 ppm (methine; rNOE), and −3.3 ppm (other aliphatic; rNOE) were compared between groups. All statistical significance was measured by the unpaired, two-tailed Student’s *t*-test with an alpha level of 0.05. All analysis was performed in MATLAB (R2016b, The MathWorks, Natick, MA).

### Oxygen consumption rate measurement

An independent measurement of the metabolic rate of the two DU145 cell types was also performed *in vitro*. Oxygen consumption rate profiles of the parental and radioresistant cells were measured (*n* = 8 wells per group) using a live cell metabolic assay platform (Seahorse XF analyzer with Cell Mito Stress Test kit, Agilent, Santa Clara, CA) in the presence of 10 mM glucose, 1 mM pyruvate, and 2 mM glutamine^56^. The cells were subjected to serial injections of 2.0 µM oligomycin, which inhibited ATP synthase, but allowed the facilitated diffusion of protons or “proton leak” across the inner mitochondrial membrane; 1.0 µM *p*-trifluoromethoxy carbonyl cyanide phenylhydrazone (FCCP), which dissipated the inner mitochondrial membrane potential and maximized oxygen consumption; and 0.5 µM rotenone and antimycin A, which inhibited complexes I and III of the electron transport chain and stopped all mitochondrial respiration. This allowed the measurement of the oxygen consumption rate due to ATP production, the proton leak, and maximal respiration and the non-mitochondrial oxygen consumption rate, respectively, so that relative metabolic rate could be compared to relative CEST and rNOE contrast (an indirect measure of metabolism) between parental and radioresistant cells.

### Data availability

The data that support the findings of this study are available from the corresponding author upon reasonable request.

## Electronic supplementary material

Supplementary Information
