# Supplementary material for: Differentiation of Normal and Radioresistant Prostate Cancer Xenografts Using Magnetization Transfer-Prepared MRI
Source: Sci Rep. 2018 Jul 11;8:10447. doi: 10.1038/s41598-018-28731-0 (PMC6041323; doi:10.1038/s41598-018-28731-0)
Supplement: Supplementary file 1 — Supplementary Information [file 41598_2018_28731_MOESM1_ESM.docx]

Supplementary Information for **Differentiation of Normal and Radioresistant Prostate Cancer Xenografts Using Magnetization Transfer-Prepared MRI**

Wilfred W. Lam, Wendy Oakden, Leedan Murray, Jonathan Klein, Caterina Iorio, Robert A. Screaton, Margaret M. Koletar, William Chu, Stanley K. Liu, Greg J. Stanisz

*T*_2_-weighted and CEST reference images with overlaid regions of interest of all the tumours scanned are shown in Supplementary Fig. S1. The analysis of three parental tumours (Supplementary Fig. S1b) was complicated by the degree of heterogeneity, rendering the simple *T*_1_ and *T*_2_ thresholding used to identify the other tumours insufficient. Therefore, these three cases were excluded. Preliminary assessment of hematoxylin and eosin stained tissue sections (data not shown) indicated a complex array of muscle, adipose, hyperchromatic hyperplasia, inflammation, apoptotic cells, and extensive fibrotic connective tissue. Radioresistant tumours also exhibited a degree of heterogeneity in *T*_2_-weighted images (Supplementary Fig. S1c); however, all presented a more uniform hyperplasia with better defined tumour margins.


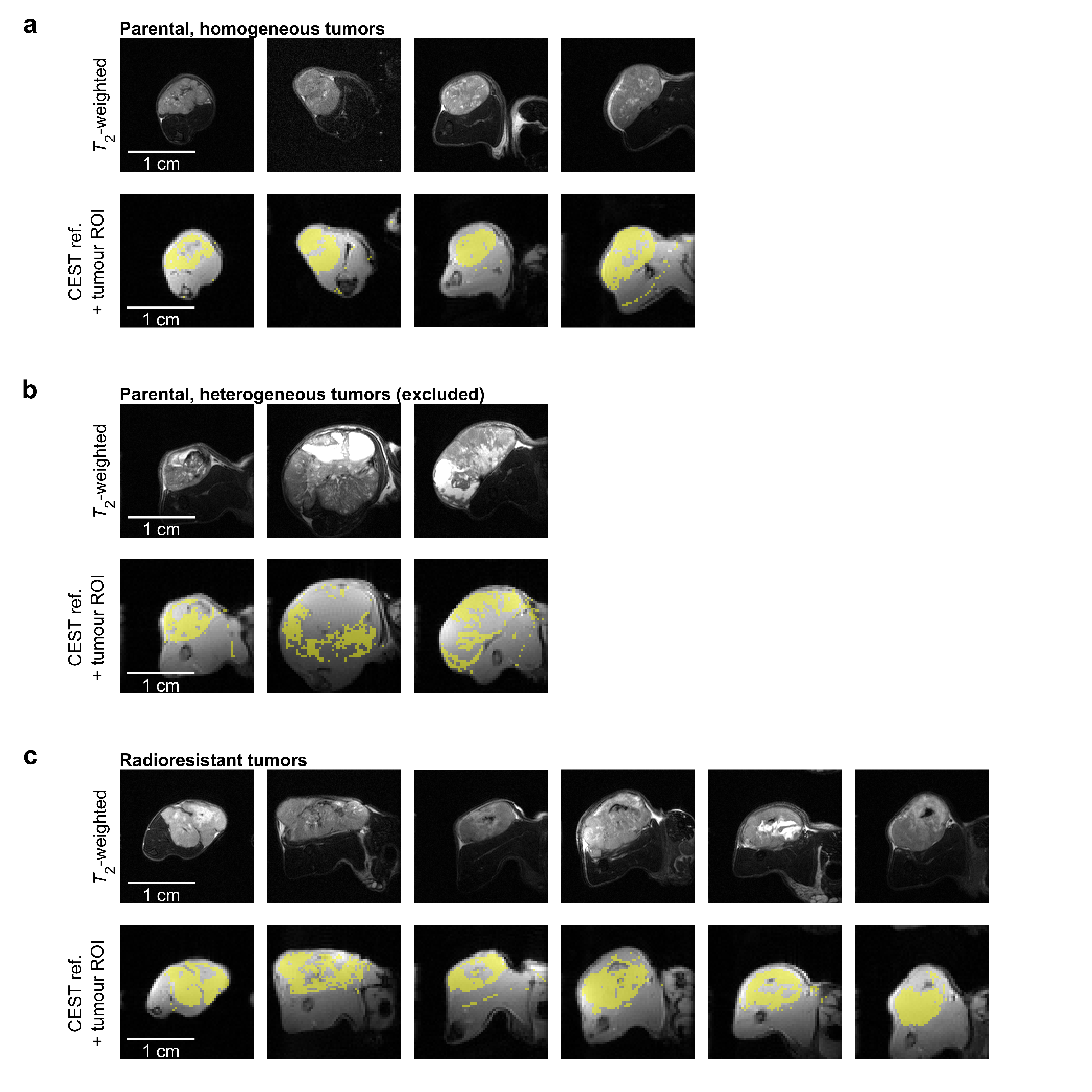

**Supplementary Figure S1.** *T*_2_‑weighted and CEST reference images (saturation *B*_1_: 0.5 µT, frequency offset: 667 ppm) with tumour region of interest (ROI) overlaid of all (**a**) homogeneous and (**b**) heterogeneous tumours, both derived from the parental cell line, and (**c**) tumours derived from the radioresistant cell line. The three heterogeneous parental tumours were excluded from analysis. All radioresistant tumours were analyzed.


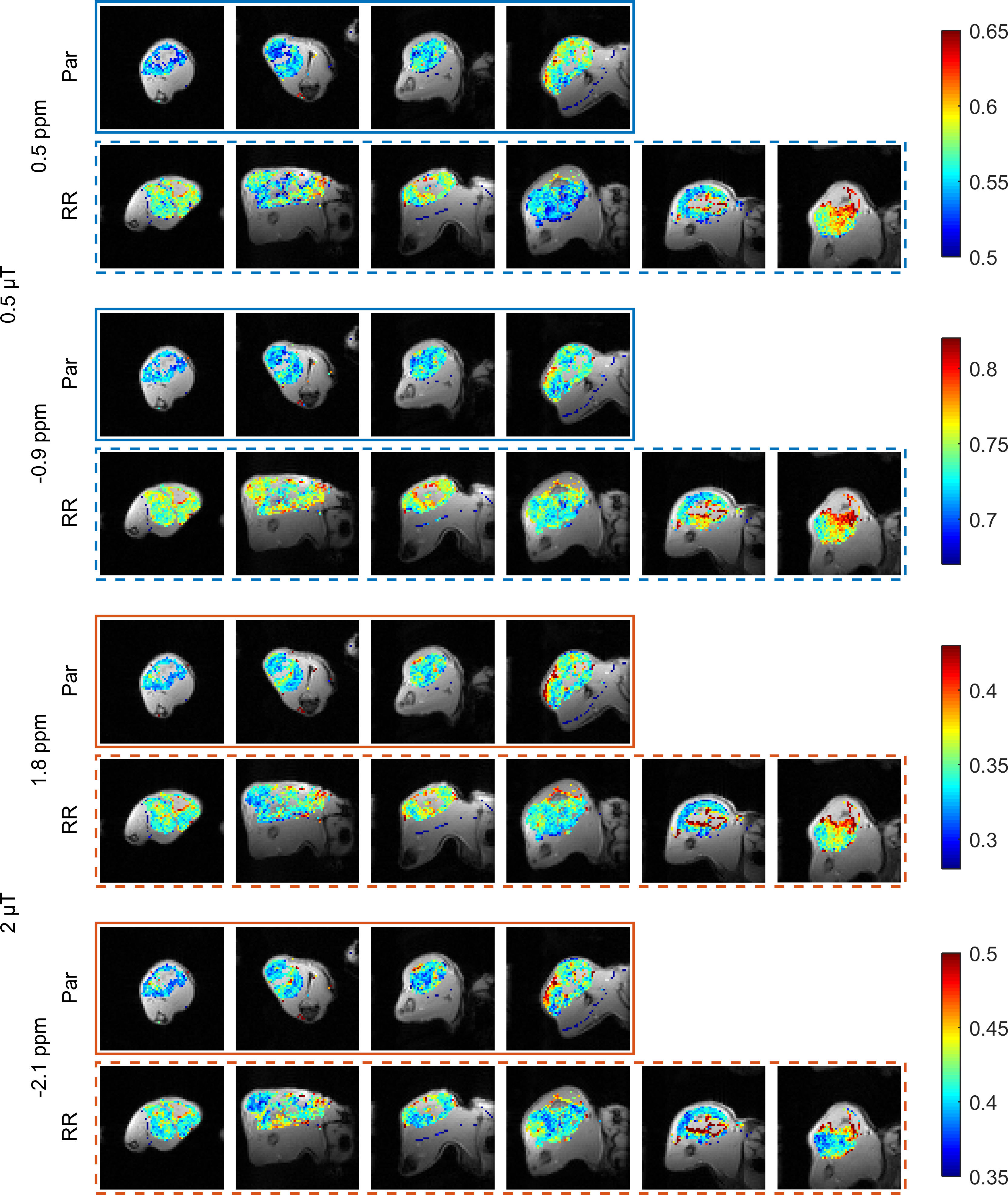


**Supplementary Figure S2.** Magnetization transfer-prepared images (with Rician noise bias and *B*_0_ correction) at the saturation *B*_1_s and frequency offsets of interest in Fig. 2 overlaid on the CEST reference images for all tumours. The colour bar limits correspond to the *y*-axis limits of the detail plots in Fig. 2a. Rectangles with solid borders indicate parental tumours and dashed border, radioresistant. Blue rectangles indicate a saturation *B*_1_ of 0.5 µT and red, 2 µT.

**
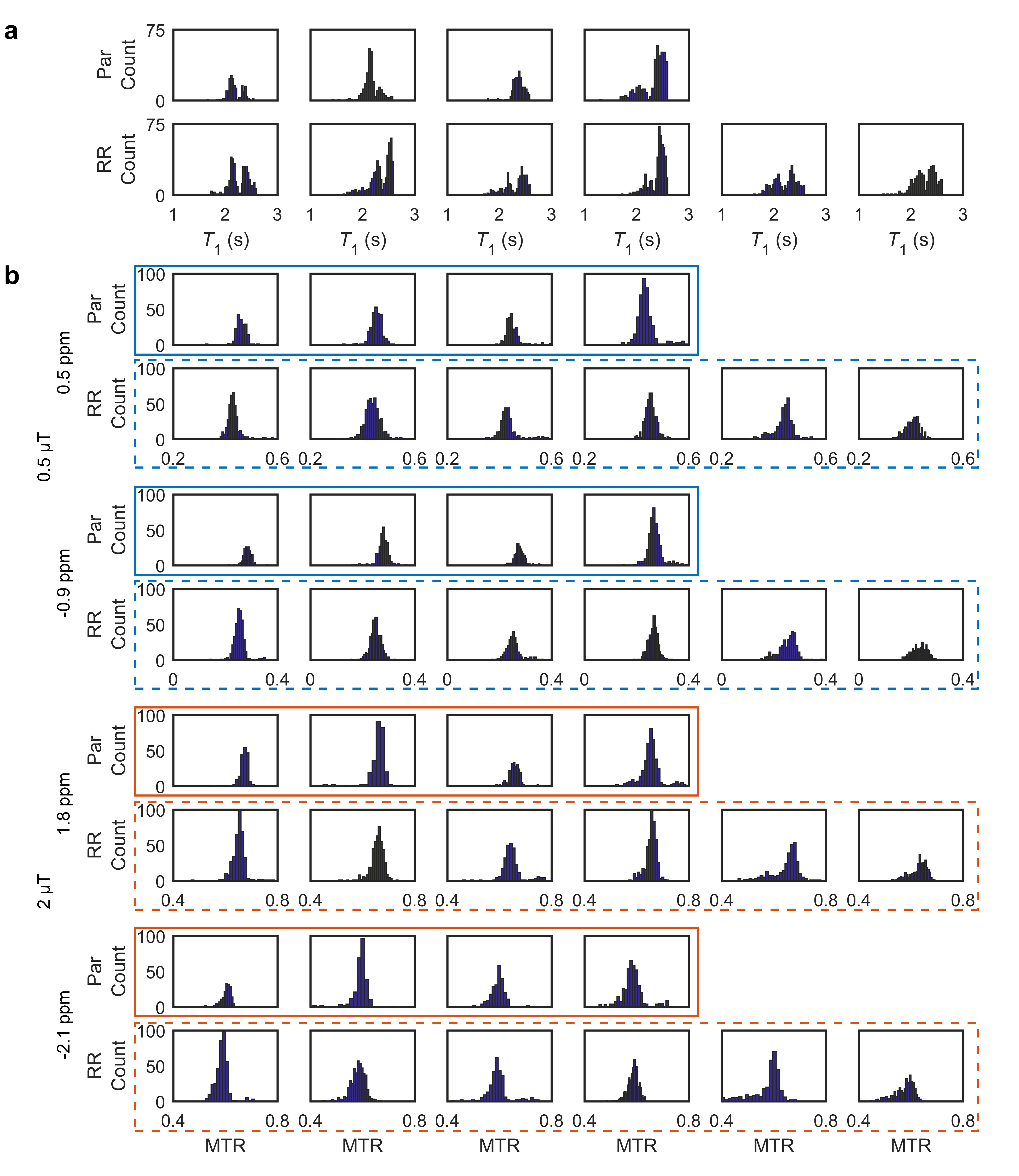

Supplementary Figure S3.** Histograms (30 bins each) of (**a**) *T*_1_ and (**b**) magnetization transfer ratio (MTR = 1 – CEST contrast) at the saturation *B*_1_s and frequency offsets of interest in Fig. 2 of the tumour regions of interest. There is intragroup and intergroup variability in *T*_1_, but no apparent pattern. There is little intragroup MTR variability and subtle intergroup variability with a saturation *B*_1_ of 0.5 µT at ‑0.9 ppm, where there is a significant MTR difference. Rectangles with solid borders indicate parental tumours and dashed border, radioresistant. Blue rectangles indicate a saturation *B*_1_ of 0.5 µT and red, 2 µT.


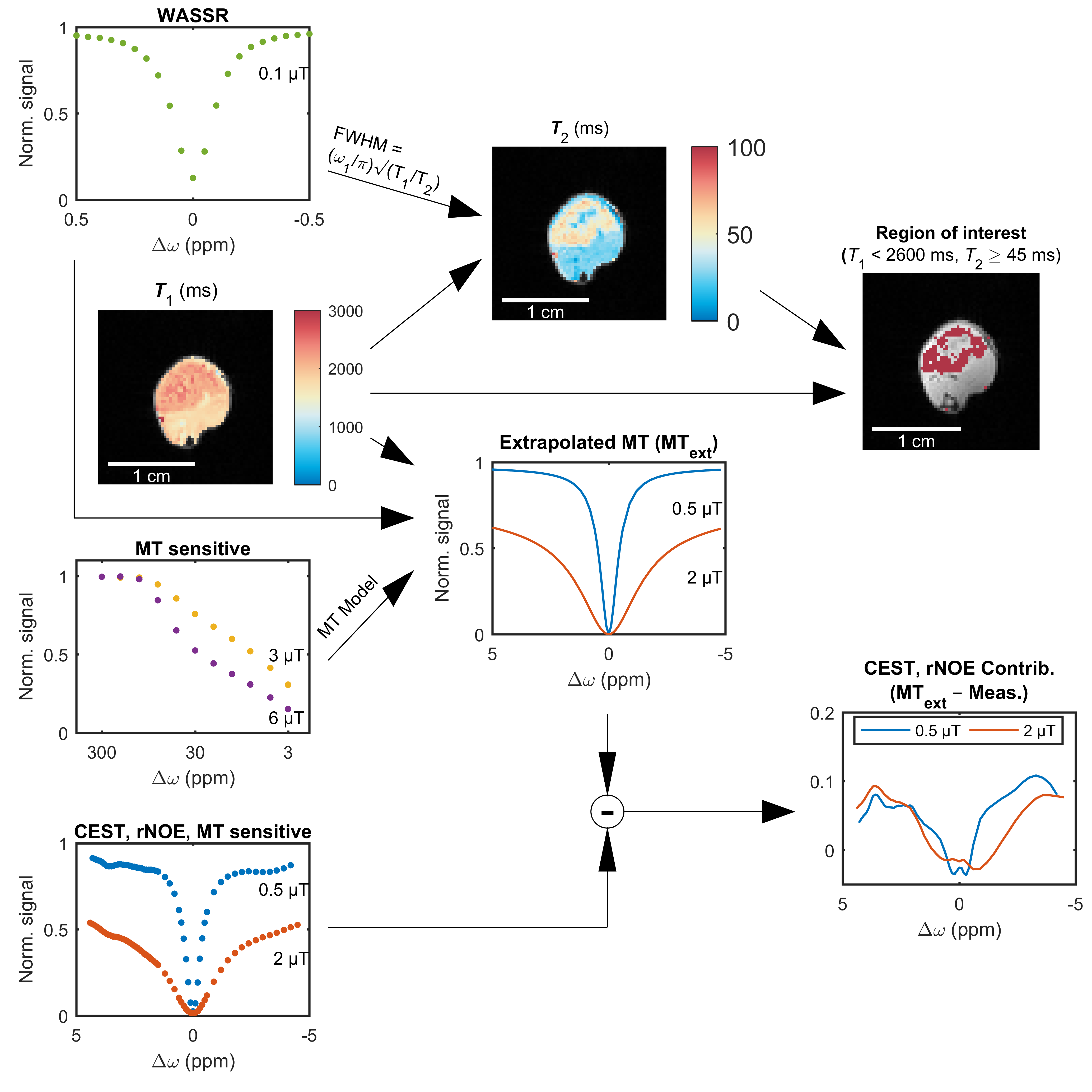

**Supplementary Figure S4.** Analysis pipeline (representative data shown). A WASSR Z‑spectrum and *T*_1_ map are used to calculate a *T*_2_ map. FWHM is the full width at half maximum. The tumour region of interest (overlaid on a Z‑spectrum reference scan, which is *T*_1_‑weighted and has isointense tumour and muscle) was defined as voxels with *T*_1_ < 2600 ms to exclude liquid and *T*_2_ ≥ 45 ms to exclude muscle and thrombus. Measured Z‑spectra with saturation *B*_1_s of 3 and 6 µT (yellow and purple points, respectively), which are mainly sensitive to the MT and water pools, are simultaneously fitted with the WASSR Z‑spectrum and *T*_1_ map to the two-pool MT model and extrapolated (MT_ext_) to saturation *B*_1_s of 0.5 and 2 µT. However, MT_ext_ does not include the CEST and rNOE contributions found in the measured Z‑spectra (blue and orange points). The difference between MT_ext_ and the measured Z‑spectra mainly reflects the CEST and rNOE contributions.

**
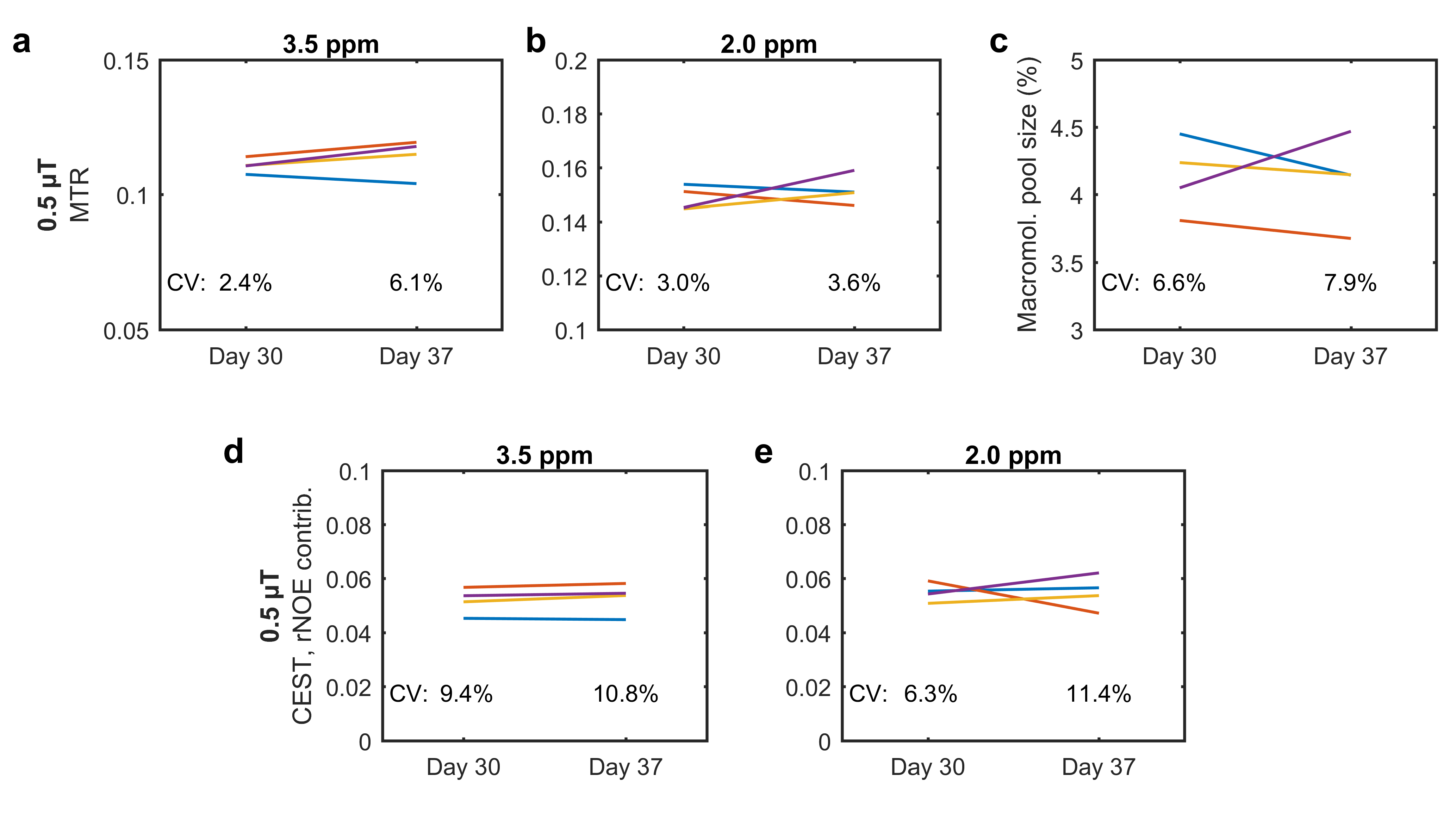

Supplementary Figure S5.** Assessment of the repeatability of the imaging protocol. Magnetization transfer ratios (MTRs) at (**a**) 3.5 and (**b**) 2.0 ppm, (**c**) the semisolid macromolecular pool size (*M*_0,B_) relative to that of water, and CEST and rNOE contributions at (**d**) 3.5 and (**e**) 2.0 ppm between radioresistant tumours (*n* = 4) scanned with a saturation *B*_1_ of 0.5 µT at Day 30 post-injection and re-scanned at Day 37. The line colour refers to the different tumours. Frequency offsets that are not expected to change over time as the tumours differentiate were chosen. All show no significant difference (*p* > 0.05). The coefficients of variation (CV; the standard deviation divided by the mean) are also indicated.


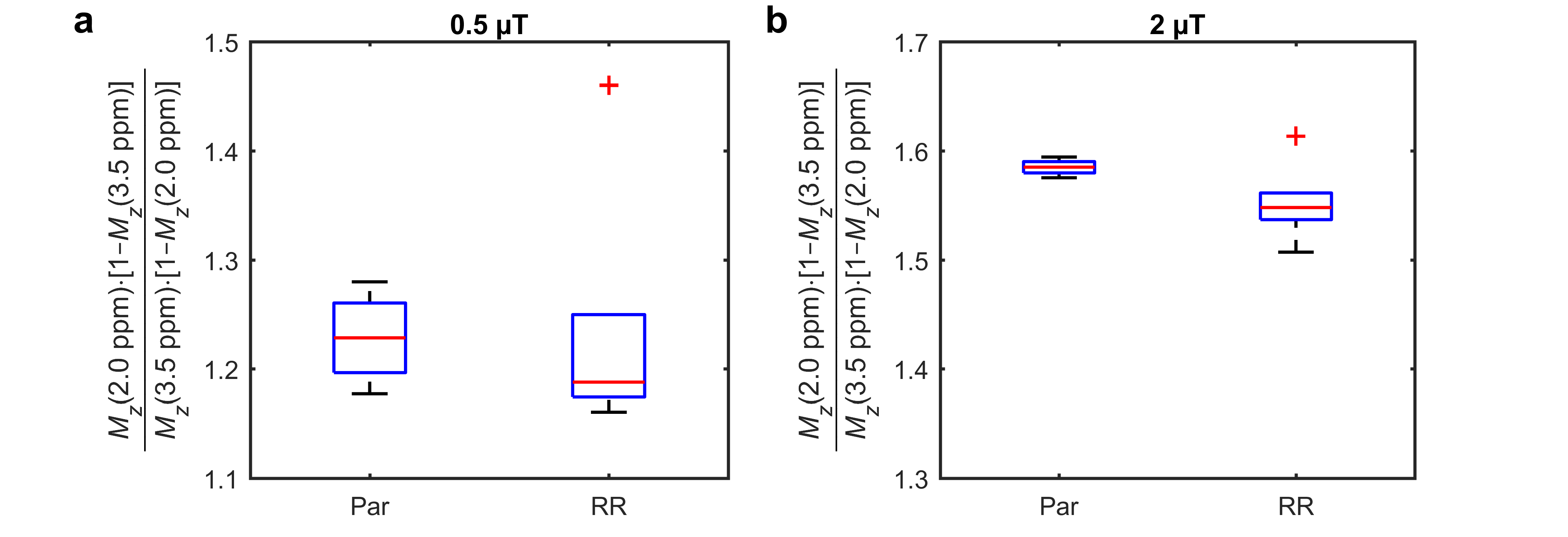

**Supplementary Figure S6.** Eq. 1 evaluated for the measured data, where *M_z_* is magnetization transfer-prepared contrast at the indicated frequency offset for *B*_1_s of (**a**) 0.5 and (**b**) 2 µT. The value of Eq. 1 varies inversely with pH, indicating that the radioresistant tumours may have a higher pH than parental ones. However, the results are not statistically significant, nor could they be translated to pH values without a calibrated standard curve.

Flip angle scale factor maps, calculated by the acquisition of a series of 3D FLASH scans (TR = 200 ms; TE = 4 ms; flip angles = 140°, 160°, …, 220°; FOV = 20 mm × 20 mm × 11 mm; matrix = 64 × 64 × 11; bandwidth = 50 kHz) and fitting to the model in Wang, J. *et al.* *J. Magn. Reson.* **182,** 283–292 (2006), are shown in below. The centre slice of the 3D volume corresponds to the slice of interest and is expected to have a good slice profile. The other slices were ignored.


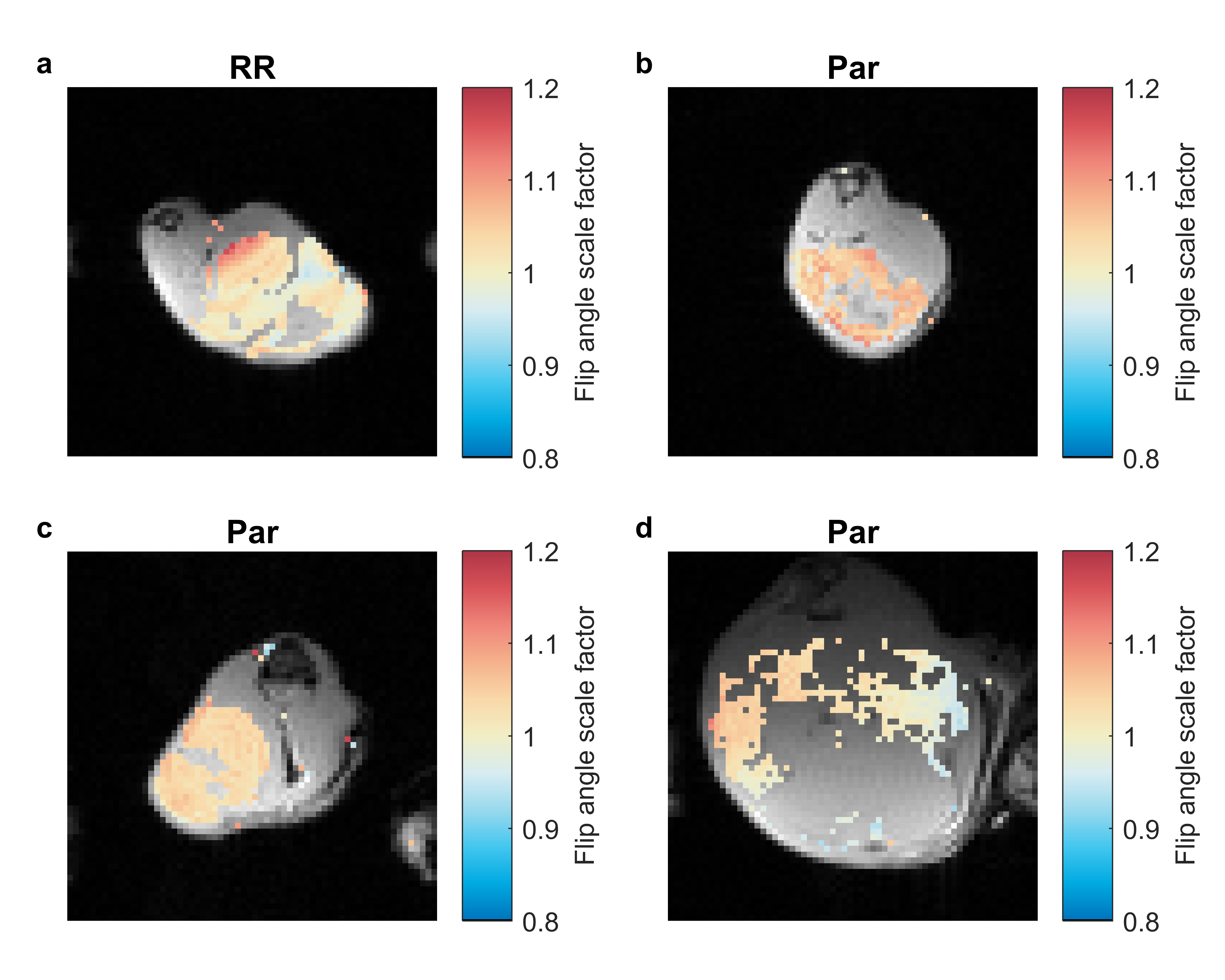

**Supplementary Figure S7.** Flip angle scale factor maps in the slice of interest in four tumours. *B*_1_ over the tumour was fairly homogeneous with flip angle scale factors of (**a**) 1.02 ± 0.04, (**b**) 1.06 ± 0.02, (**c**) 1.04 ± 0.02, and (**d**) 1.01 ± 0.03.
